# Improving the smart care service system for older adults: an emotional experience evaluation framework in the Chinese community

**DOI:** 10.3389/fpubh.2025.1614150

**Published:** 2025-10-01

**Authors:** Yuexuan Wu, Zijian Liu, Xuemei He, Xianru Shang

**Affiliations:** School of Art and Design, Shaanxi University of Science and Technology, Xi’an, China

**Keywords:** smart care service system, PAD emotion theory, strategy research, emotional experience evaluation, China’s community settings

## Abstract

**Objectives:**

Given the rapid growth of the older population, an emotional experience evaluation system is proposed to quantitatively analyze the emotional experiences of older adults within the smart care service system in the current Chinese community settings.

**Methods:**

The study is informed by the Pleasure-Arousal-Dominance (PAD) emotion theory and guided by service design thinking. Qualitative research methods were employed to gather information about older adults within the target community. By mapping service scenario interaction touchpoints, the research team deconstructed, clustered, and transformed the system service touchpoints. Evaluation indicators were set from three aspects: smart devices, online software, and offline services, to construct an emotional experience evaluation system. Using quantitative research methods, with user emotional valence as the variable, the study conducted measurement experiments on the emotional experience evaluation of older users, obtaining measurement results from two dimensions: emotional experience tendencies and emotional requirement assessment.

**Results:**

We organized the results of emotional experience evaluation data, analyzed the indicator data of each part of the system through visual analysis graphics, and proposed improvement strategies for the three structures of the smart care service system.

**Conclusion:**

Care for older adults is facing multifaceted challenges, our research results have obtained the emotional tendencies of older adults toward each indicator of the system. In addition, we have obtained the judgment results of emotional demand for each indicator, analyzed the reasons from both emotional and demand dimensions, proposed service strategies to improve the system, and facilitated the implementation of a circular economy model in community care services for older adults.

## 1 Introduction

The growing global population is placing significant strain on the economic and social systems of countries worldwide. How to provide better care service experience for older adults is an urgent social problem that needs to be solved globally. Furthermore, advances in modern medicine continue to increase the life expectancy of older adults, resulting in a growing demand for nursing services (1). The gradual development of “Humanization of Care” and its fundamentals are anticipated to provide healthcare institutions with efficient nursing guidance (2). Globally, ongoing reforms of national healthcare systems are providing healthcare services that are more accessible to older users and gradually bringing services into their homes. These reforms tackle challenges in older populations, chronic disease growth, and enduring social inequality (3). In various case studies, the transformation of nursing structures is closely related to the rapid development of digital technology. Recently, the proliferation of products of the technological revolution, and for instance, artificial intelligence, the Internet of Things, big data and cloud computing, has given a great impetus to the evolution of smart care services (4), about which there are different histories of development in both developing and developed countries. In the 1950s, the British Trust Fund first introduced smart care for older adults, a novel model that has since gained public visibility (5). The concept of the smart home was first introduced by the American Association of Builders in 1984, referring to residences equipped with interactive intelligent technologies (6). In Sweden, e-Home care system employs Information and Communication Technology (ICT) to provide home-based services such as monitoring, reminders, information delivery, and social interaction (7).

In China, given the demographic challenges and large population base described above, the integration of smart home design, to build and delivery with smart community building is regarded as a crucial approach for establishing sustainable and inclusive models for older populations (8). Smart homes for older adults’ care emerged in China in 2008 and have since evolved through four distinct developmental stages, namely the seed stage (2008–2011), the start-up stage (2012–2014), the development stage (2015–2016), and the popularization stage (2017–2019) (9). In 2021, China’s Ministry of Industry and Information Technology, Ministry of Civil Affairs, and National Health Commission jointly launched the “Smart Health Care Industry Development Action Plan (2021–2025)” to promote the sector’s growth (10), and the content of the action plan indicates the country’s determination to promote social informatization and digitization. The smart home model promotes the use of innovative technologies in older adults’ care, including virtual support groups, video-conferencing, and digital health records for support; sensors, wearables, and telehealth for safe independent living; and interactive robots such as humanoid, rehabilitation, and companion robots for mobility and cognitive assistance (11). As a result, the smart home care market is poised for substantial growth in the coming decades due to increasing demand. Nevertheless, multiple barriers hinder its widespread adoption, with unbalanced resource ratios and imperfect information integration affecting the unification of smart care systems. Liu et al. (12) examined the interactive design of older adult care service applications within smart care communities, deeply analyzed the physiological and psychological needs of older users, identified limitations in existing applications, and pinpointed key factors for enhancing interaction design in such services. Zhao et al. (5) first combined the theory of social emotional choice to achieve coordination and optimization of the dual channel supply chain for smart older adult care services. Based on service design theory, Wen et al. (13) focused on analyzing the lifestyle and nursing service models of older adults in nursing homes, and proposed service strategies. Xu et al. (14) enhanced the smart older adult care service platform using gray relational analysis and Fuzzy-QFD, adapting it to better align with older adults’ preferences.

The smart care service system for older adults is a new type of care service model that depends on the community and associated “treatment, recuperation, and nursing” organizations (15). It differs from the traditional care model in that it offers older adults basic medical protection. China’s care service model for older adults has advanced from physical technology to intelligent technology with the support of big data, the Internet of Things, and related smart technologies. (16, 17). With intelligent nursing as its foundation, the intelligent recreation and nursing service system leverages information technology and other contemporary science and technology to support the management and life, including older safety and security, medical care and health, rehabilitation and health care, leisure and recreation, and learning and sharing (18–20). In terms of resource integration, smart healthcare effectively utilizes the government’s environmental supply for community older adult care and the establishment of basic medical structures, reducing the pressure on the social medical system (21, 22). In terms of service demand, the smart health care service system can meet the basic daily life care, medical rehabilitation care, social activity participation, spiritual comfort services, and value contribution needs of older adults (23, 24). As one of the important models, the formation of China’s care service system for older adults has more multi-mode and open characteristics compared to home-based and institutional older adults’ care. (25). In 2020, the country launched a project to establish pilot institutions for remote collaborative older adult healthcare and intelligent care (9). The openness and inclusiveness of the market have attracted multiple social forces to join.

Our findings demonstrate that technological advancements have transformed older adults’ care from externally assisted models to home-based service delivery. Enabled by assistive technologies, an increasing proportion of older adults now opt for home care. This represents an open, cyclical service ecosystem rather than an isolated care model. Current research has substantially advanced the evolution of smart care systems within community settings, with extensive studies addressing older adults’ psychological and physiological requirements. To enhance these systems further, we emphasize emotional experience as a critical research focus. Adopting a service design and user experience approach, we investigate older users’ emotional responses to smart care systems through targeted variable measurement. It is worth considering that the existing smart care system is a comprehensive layout. To better understand older adults’ actual care needs, a systematic deconstruction is required, analyzing the touchpoints between users and the system through coded quantitative methods. This approach enables the formulation of corresponding improvement strategies for each part of the system, thereby better promoting the advancement of smart care for older adults.

## 2 Theoretical framework

### 2.1 Theoretical basis

Emotions occur within the subject and are emotional reactions triggered by external stimuli entering the individual’s psychological world. Users’ assessments of the whole service experience might be directly impacted by emotional shifts. Scholars are paying more attention to the subjective issue of how to objectively use emotions in the form of quantitative data in real-world service experiences (26–28). The measurement of emotional experience still mostly uses the category and dimension methods that are frequently used in psychology to measure emotions or combines the two approaches for measurement, much like the research methodologies used in other fields (29). Users’ emotions are categorized using the categorization approach, and there are two popular classification schemes. One is to broadly classify users’ emotions into positive and negative emotions, and the other is to classify users’ emotions into basic emotions and compound emotions (27). The dimensional emotion model refers to a multi-dimensional model of affects, such as the PAD emotion model (30).

The PAD emotional theory, proposed by Mehrabian and Russell in 1974, is a multidimensional model for measuring emotions. It posits that human emotional experiences are not a unitary physiological manifestation, but rather a complex and multifaceted phenomenon (30). This model classifies human emotions into three fundamental dimensions: pleasure, arousal, and dominance. Pleasure (P) reflects an individual’s positive or negative affective response to information. Arousal (A) corresponds to the intensity of physiological activation elicited by external stimuli. Dominance (D) describes the perceived level of control over the information or situation. The PAD emotion theory is an emotional measurement method based on psychological responses. It has strong operability in emotional computing, which stems from the spatial nature of the theoretical model. By quantifying and establishing the positioning and relationships of several emotional categories in the emotional space, it can calculate the difference between user experience emotions and basic emotional values, thereby determining emotional tendencies. Current research primarily focuses on the user emotional experience measurement stage. Huang et al. (31) explored seven main “acoustic features” and their differences in physical features related to gender identification and manifestation of affective states in the PAD emotion model during speech interaction. Zhao et al. (32) combined the proposed emotion model with the Pleasure-Arousal-Dominance (PAD) emotion model and the Five Factor Model (FFM) to evaluate user emotions of three information system features (user interface, interaction quality, and service environment) in mobile libraries. Xu et al. (33) applied the PAD emotion theory to explore the impact of online comment emotions on perceived comment usefulness built upon these three basic emotional dimensions.

From the above, the PAD emotion theory used in the evaluation and measurement of service system has significant application value, so our study applied this theoretical framework to quantitatively assessed emotional experiences within smart care systems, generating empirically validated outcomes.

### 2.2 Application of PAD emotion theory in smart care service system

Smart care service systems supply older adults with more personalized, convenient, and intelligent attributes of online and offline interaction in the model of older adults’ care. Our scholars have proposed that the basic operational structure of the system consists of three major segments: smart devices, online software, and offline services (34, 35). Smart devices mainly refer to intelligent recreational products, including wearable health management devices, portable health monitoring devices, self-service medical check-up devices, home smart care monitoring devices, home service robots, etc. Online software mainly includes catering service apps, life service apps, shopping service apps, medical service apps, travel service apps, entertainment service apps, etc. Offline services mainly include meal assistance services, life care services, mobility assistance services, home medical care services, offline medical care services, mental comfort services, emergency assistance services, etc. Smart devices are the foundation, through the collection of older adults’ living habits, health status, and other life demand data input to the online software platform, the online software platform for offline service circle reasonable distribution, output accurate services, forming a closed loop, and realizing the matching of older adults’ services and demand.

**Figure 1:**
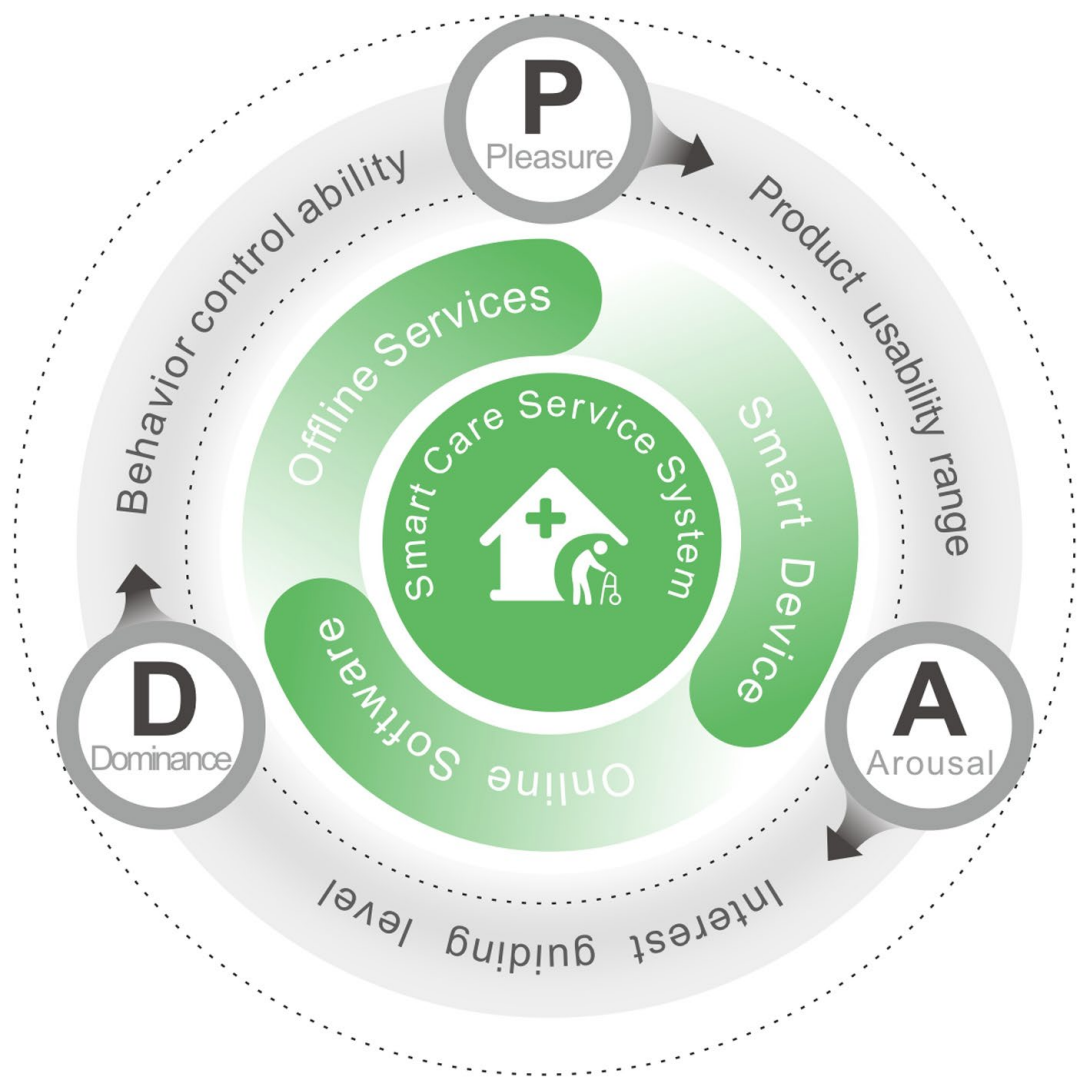
The PAD emotion theories for description of smart care service system.

The emotional experiences of older adults, who are the target audience for the smart care service system, are able to naturally mirror this system’s current issues. The degree of pleasure can indicate how user-friendly and easy the system is to use. High levels of pleasure allow the user to think and make decisions with ease, choose the best method to finish the task, and have a positive experience. It is easier to focus and become drawn to the product and interest when the user is experiencing a specific level of arousal or excitement. However, prolonged and excessive arousal can lead to mental fatigue due to sustained high concentration. Dominance is a measure of how controllable a product or service is for the user, the more dominance there is, the lower the user’s mental effort and knowledge acquisition burden, and the more likely the user is to follow the system’s presetting to function correctly, i.e., the higher the degree of service completion (32). Therefore, the PAD emotion theory can obtain quantitative data of user’s emotional experience in this three aspects of the product usable range, interest guidance level, and behavioral control ability, respectively, which are highly consistent with the direction of the evaluation of the experience of the smart devices, online software, and offline services that make up the smart care service system. We use PAD emotional theory to describe the content of this system, forming a circular model as shown in Figure 1, this indicates that the PAD emotion theory can be employed for emotional experience evaluation of the smart care service system. Furthermore, in our study, we not only measured the emotional experiences of older adults, but also assessed their emotional needs.

## 3 Materials and methods

### 3.1 Emotional experience process design for smart care service system

1. Establishment of the emotional experience evaluation index system. We obtain older adults’ information through participatory field research methods and user behavior tracking technology, deconstruct the smart care service system, simulate older adults’ behavior through the service scenario interaction touchpoint map which is a commonly used tool in service design, used to simulate user interactions with scenarios, and cluster service touchpoints. Under the guidance of experts, the research team conducted a qualitative analysis of service touchpoints, converted these into corresponding emotional metrics, and established a logical evaluation index system.
2. Process of the emotional experience evaluation measurement experiment. We first collected the experimental data among the subjects, on the one hand, we carried out the emotional potency analysis, poured the experimental data into the analysis software to get the mean value of emotional evaluation, and then got the emotional experience bias dimension after the formula calculation. On the other hand, the secondary division was carried out under the support of Maslow’s hierarchy of needs theory (36), we also determined the needs of these data indicators.
3. Evaluation results and improvement strategies of the emotional experience. Using visual analysis graphics, the research team arranged the scale data we had collected and examined each of the system’s three structures separately. In order to maximize the current smart health care system and improve the emotional experience for older adults, we proposed improvement strategies for the three-part system structure and addressed the causes of user emotional experience bias and demand analysis from the viewpoints of comparative evaluation and factor analysis.

**Figure 2:**
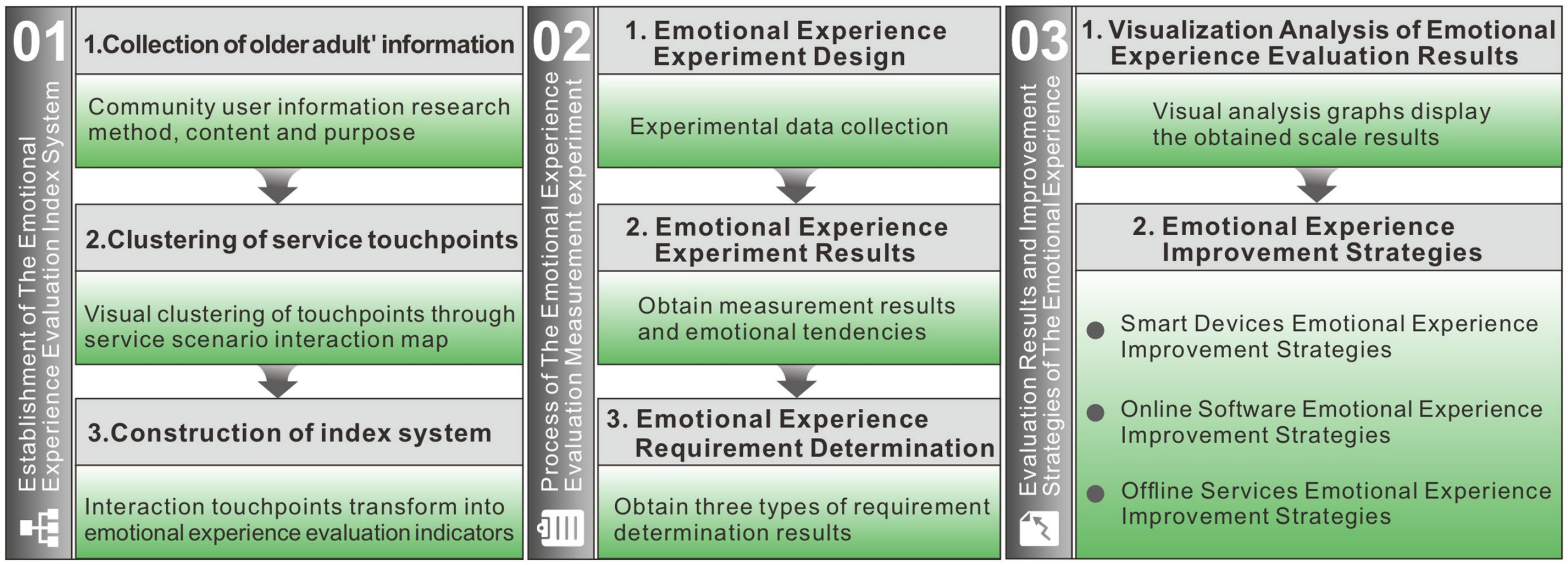
Emotional experience evaluation process.

The overall experimental process is shown in Figure 2. The three main parts of the study, from the establishment of emotional experience evaluation system to the experimental process and the analysis of results, are briefly summarized in each section.

### 3.2 Collection of older adults’ information

According to the national demonstration initiative for older-adult-friendly communities issued by the Chinese Health Commission, field research was conducted on three of the communities listed in Xi’an, including Dongfang Community in Hansenzhai Street, Xincheng District, Qianhu Community in Jiapo Street, Yanta District, and Fengye Huizhi Community in Zhangba Street, High tech Zone. The advantages of these three innovative older care model communities over traditional ones include a clean and safe living environment, easy access to health and medical services, a high level of technological innovation for older adults’ care, and widespread social interaction. At present, this community collaborative open smart care service model is progressively gaining popularity in large and medium-sized cities.

Basic data about the community’s older adults, their lifestyle choices, and a survey asking about their satisfaction with the software, product, and life service make up the majority of the research content. Observation, action tracking, questionnaire research, semi-structured interviews, and other techniques are all part of the participatory research approach. The purpose is to explore the existing problems of the smart care service system through the tracking process of some behavioral contacts, processes, and experiences of older adults’ life trajectory, to assemble, screen, and quantify the service contacts of behaviors, and to further put forward the service design strategies, and the specific research methodology, content, and purpose are shown in Table 1.

**Table 1:** Community user information research methods, content and purpose.

**Research methods**	**Content**	**Purpose**
Observation method	Concentrate on choosing locations such as community dining sites for older adults, rehabilitation and physiotherapy rooms, community health service centers, nearby hospitals, supermarkets, stores, community activity centers, academy for older adults, etc., and record the process of older people’s access to products, software, and services.	Knowledge of the habits of older adults and the types of products and services to which they are exposed.
Action tracking	Researchers observed and tracked older adults’ behavioral patterns throughout the day without disrupting their daily routines, while recording data in proximity.	Collection of activity routes for older adults as well as operation of products and frequency of services received.
Questionnaire survey	A structured questionnaire was used, which mainly included: basic living conditions of older adults, medical and nursing needs, product use, life service user experience satisfaction survey, and suggestions for older adults’ care development model.	Master the basic older adults’ care situation and deeply explore the implicit needs of older adults for product use, software operation, and life services.
Semi-structured interview	Thirty-five older adults with typical behavioral trajectories were selected for in-depth interviews that focused on the experiences and needs of each behavioral activity.	The behavioral activity patterns of older adults were analyzed, and behavioral touchpoints were divided to do quantitative analysis.

### 3.3 Clustering of service touchpoints

To establish an emotional experience evaluation index system for the smart care service system of our research community, according to the research method in Table 1, user information of older adults in three aspects, smart devices, online software, and offline services, are obtained, and the information is integrated and categorized through the service scenario interaction touchpoints map. Our system leverages existing infrastructure to establish a modular information network for older adults at the community level, and the simulated real-life three-dimensional model is used as a map to grasp the operation of each area in real-time through the big data of older adults activities, and the information is integrated and classified through the interactive touchpoints map of the service scene.

**Figure 3:**
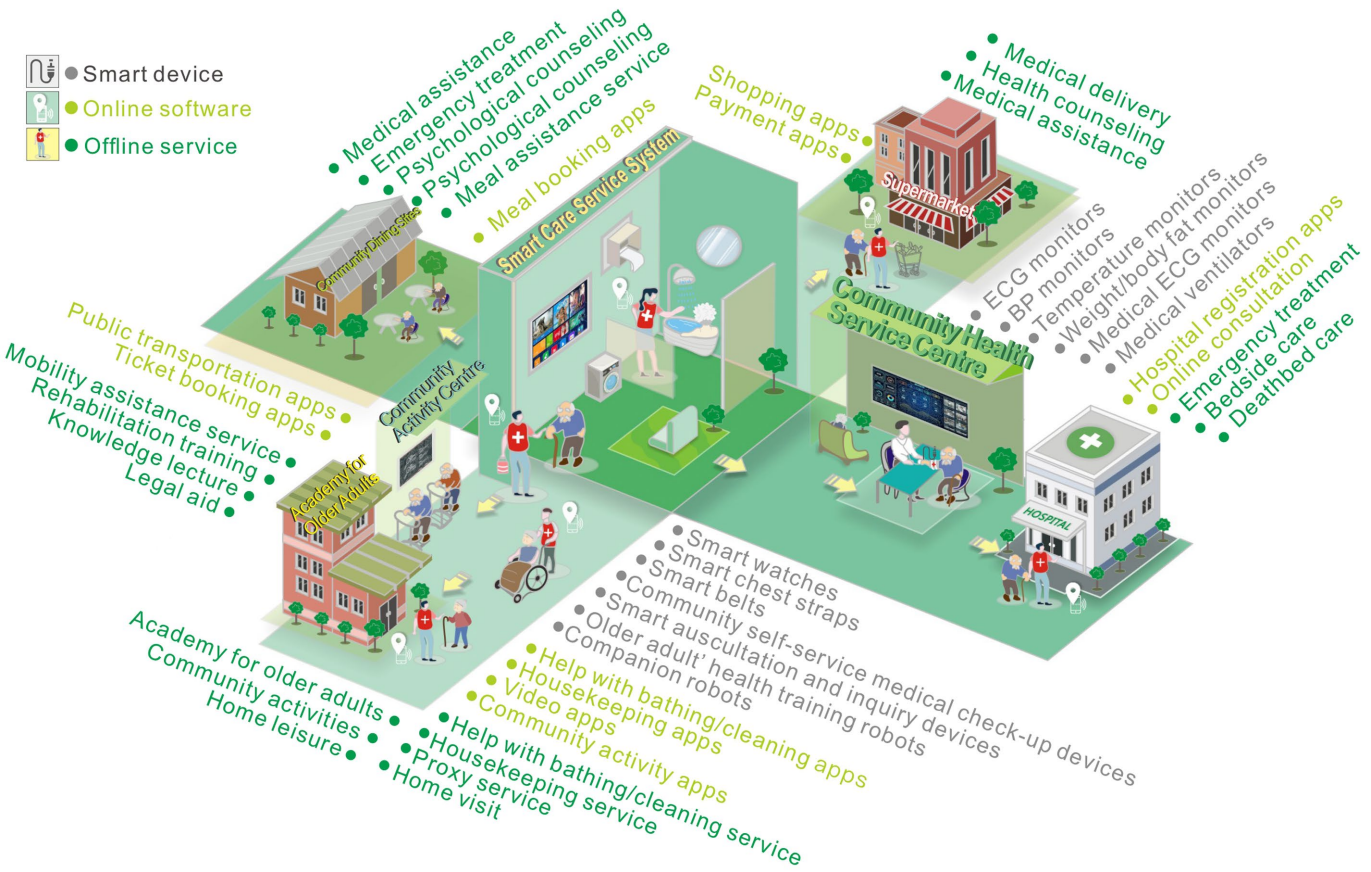
Smart care service scene interaction touchpoint map.

In the service design research method, the service scenario interaction touchpoint map can simulate the basic operation structure of the service system and display the service process intuitively and clearly. This study applies the service scenario interaction touchpoint map to visualize and cluster each part of the contacts in the system in the research results, as shown in Figure 3, and the research team carries out a comprehensive analysis from the results of field research and lists multi-frequency interaction contacts in the three parts of the smart care service system.

### 3.4 Construction of index system

The research team conducted a qualitative study on the high-frequency interactive service touchpoints obtained through expert guidance and group discussions and merged analogous interaction points into emotional experience evaluation indicators. In terms of smart devices used by older adults, devices of the same category are grouped together and receive five emotional experience evaluation indicators, namely wearable health management devices, portable health monitoring devices, self-service medical check-up devices, home smart care monitoring devices, home service robots. In terms of the use of online software by older adults, online software with the same functions is classified into one category, and six emotional experience evaluation indicators are obtained. The functional categories include catering service apps, life service apps, shopping service apps, medical service apps, travel service apps, entertainment service apps. In terms of older adults enjoying offline services, those with the same service direction are classified into one category and receive seven emotional experience evaluation indicators, including meal assistance services, life care services, mobility assistance services, home medical care services, offline medical care services, mental comfort services, emergency assistance services.

Therefore, the 46 interaction contacts in the service scenario interaction touchpoint map of the service scene are converted into 18 emotional experience evaluation indexes to establish the emotional experience evaluation index system of this smart care service system, complete index coding, explain indexes to facilitate the subsequent emotional experience evaluation measurement experiments, and the older users can correctly understand the index meanings, as shown in Table 2.

**Table 2:** Emotional experience evaluation index system of smart care service touchpoints.

System structure	Touchpoint	Index	Indicator Interpretation	Code
Smart devices	Smart watches	Wearable health management devices	Wearable smart devices for managing health data (including smart watches, chest straps, belts, clothing built-in devices, etc.)	S_1_
	Smart chest straps			
	Smart belts			
	ECG monitors	Portable health monitoring devices	Portable and mobile health monitoring devices (Monitor multi-parameter health data such as ECG, BP, temperature, weight/body fat, etc.)	S_2_
	BP monitors			
	Temperature monitors			
	Weight/body fat monitors			
	Community self-service medical check-up devices	Self-service medical check-up devices	Self service medical check-up devices, smart auscultation and inquiry devices provided by the community	S_3_
	Smart auscultation and inquiry devices			
	Medical ECG monitors	Home smart care monitoring devices	Home smart care monitoring devices (including medical ECG monitors, medical ventilators, etc.)	S_4_
	Medical ventilators			
	Older adults’ health training robots	Home service robots	Home friendly service robots for older adults	S_5_
	Companion robots			
Online software	Meal booking apps	Catering service apps	Online food ordering apps	S_6_
	Help with bathing/cleaning apps	Life service apps	Household cleaning service apps	S_7_
	Housekeeping apps			
	Shopping apps	Shopping service apps	Online shopping service apps	S_8_
	Payment apps			
	Hospital registration apps	Medical service apps	Online medical apps (including hospital registration apps, online consultation, etc.)	S_9_
	Online consultation			
	Public transportation apps	Travel service apps	Online travel service apps	S_10_
	Ticket booking apps			
	Video apps	Entertainment service apps	Leisure and entertainment services apps	S_11_
	Community activity apps			
Offline services	Meal assistance services	Meal assistance services	Home service for meal assistance	S_12_
	Help with bathing/cleaning services	Life care services	Household life care services (including help with bathing/cleaning service, housekeeping service, proxy service, etc.)	S_13_
	Housekeeping services			
	Proxy services			
	Mobility assistance services	Mobility assistance services	Outdoor travel services for the older	S_14_
	Home visit	Home medical care services	Home medical auxiliary nursing services (including home visit, emergency treatment, medical delivery, bedside care, deathbed care, etc.)	S_15_
	Emergency treatment			
	Medical delivery			
	Bedside care			
	Deathbed care			
	Health counseling	Offline medical care services	Offline medical care services (including health counseling, rehabilitation training, knowledge lecture, psychological counseling, etc.)	S_16_
	Rehabilitation training			
	Knowledge lecture			
	Psychological counseling			
	Medical assistance			
	Academy for older adults	Mental comfort services	Older adults’ mental comfort activities (including academy for older adults, community activities, home leisure, psychological counseling, etc.)	S_17_
	Community activities			
	Home leisure			
	Psychological counseling			
	Emergency treatment	Emergency assistance services	Professionals help emergency services	S_18_

## 4 Experiments and results

### 4.1 Emotional experience experiment design

Derived from field research findings, our research team conducted an emotional experience questionnaire survey experiment on 28 older users (16 males and 12 females) in a semi-structured interview target using random sampling. The age range of the participants was between 62–85 years old, all participants engaged in the experiment voluntarily and demonstrated a comprehensive understanding of its procedures. During the interview experiment, members of our research team explained the questions to the participants and explained the questionnaire scoring criteria. To mitigate the influence of a limited sample size on experimental outcomes, participants were selected based on their prior operational familiarity with a range of smart devices, online applications, and offline services. For example, the tested older adults usually use wearable electronic devices during the process of home-based care. They have some experience in health monitoring and have suggestions for improving product functions. They usually come into contact with a lot of online software and have a certain cultural foundation, which allows them to have more communication with online and offline services. These choices can ensure the emotional experience preference results of the target users toward various structures of the smart health care service system.

**Figure 4:**
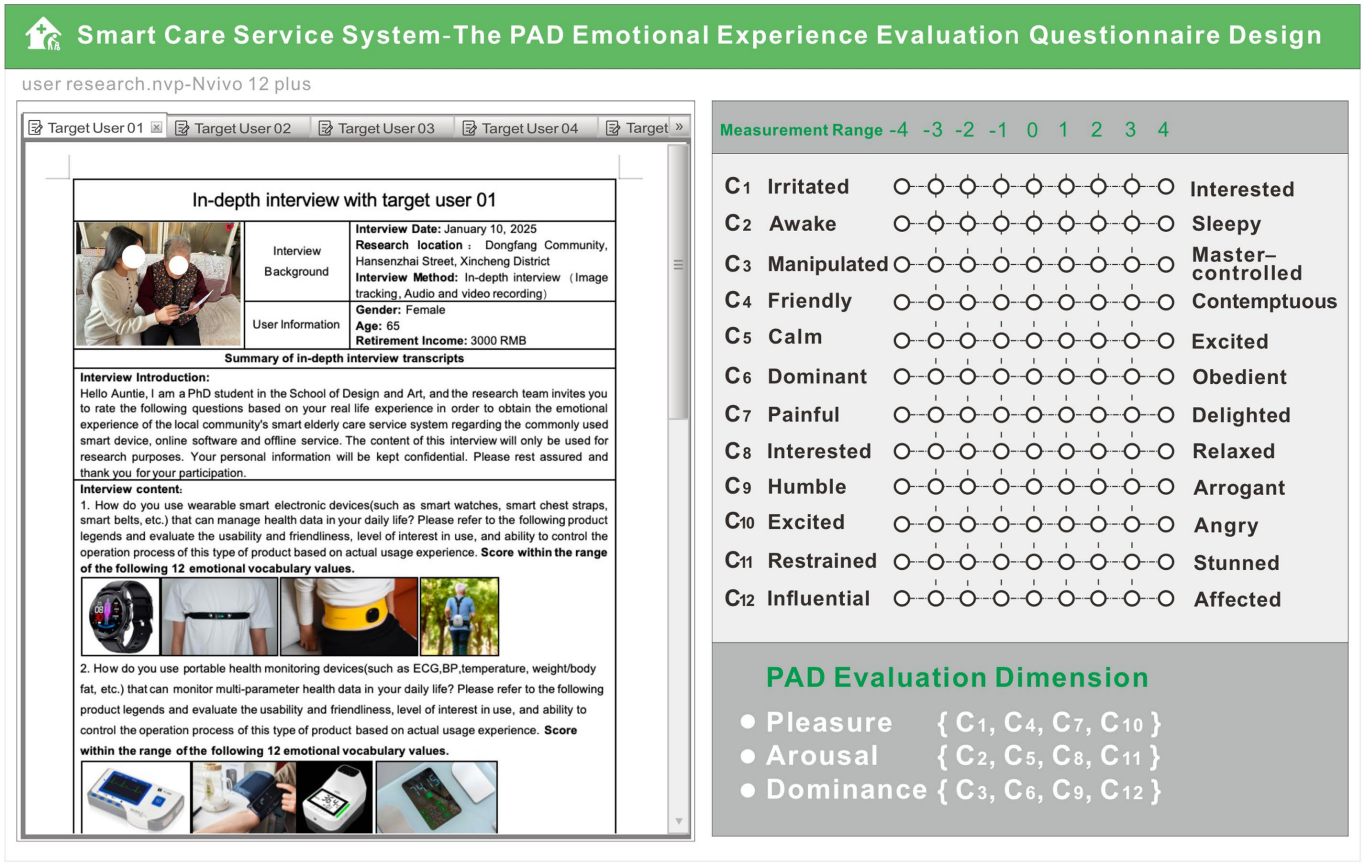
Sample smart care service system emotional evaluation questionnaire.

Specific questionnaire content was designed for the emotional experience evaluation index system of the smart care service system and combined with PAD emotion scale. To fit the emotional characteristics of Chinese people, the Institute of Psychology of the Chinese Academy of Sciences formulated the Chinese version of the PAD standardized scale as a user-multidimensional emotion measurement tool based on a multidimensional emotional space model, which adopts a nine-point scoring mechanism and is arranged in a bipolar manner according to ±4. Under each emotional dimension, four pairs of adjective groups can reflect the human body’s emotional response and have opposite semantic meanings, emphasize the degree of emotional contrast to help users choose their preferences. Each set of emotional semantics is generated by experts and serves as a fixed reference phrase. An example of the design of the questionnaire and task scale is shown in Figure 4. Pleasure includes C_1_, C_4_, C_7_, C_10_; arousal includes C_2_, C_5_, C_8_, C_11_; dominance includes C_3_, C_6_, C_9_, C_12_, and the scoring level is directly proportional to the emotional validity. The left side of the figure shows a thumbnail example of the questionnaire processor, while the right side shows the form used to score evaluation indicators during the survey process. During the experiment, the research team first asked about the basic usage of 18 survey indicators according to the questionnaire content, and then asked older users to score 12 emotional comparison phrases based on their overall usage experience. The acquired user interview results were further transformed into textual information imported into the qualitative analysis software Nvivo12.0, and the scoring results were analyzed by SPSS software.

### 4.2 Emotional experience experiment results

Following software analysis to solve the measurements for the mean value, Table 3 displays the indicators’ results.

**Table 3:** PAD emotional indicator measurement results.

Measurement indicators	P				A				D			
	C1	C4	C7	C10	C2	C5	C8	C11	C3	C6	C9	C12
S_1_	2.81	−2.65	2.57	−2.79	−1.37	1.80	−1.21	2.02	1.36	−2.13	1.05	−0.90
S_2_	2.95	−2.88	1.75	−0.10	−0.50	−1.68	1.34	−0.05	1.27	0.05	−0.08	−2.86
S_3_	−0.86	0.77	−0.05	0.12	0.05	1.64	−1.02	−0.75	−1.95	−0.33	0.20	0.67
S_4_	1.75	−0.15	−0.05	0.13	1.54	−1.66	−1.05	−0.86	−2.05	0.29	−0.05	2.95
S_5_	1.83	−2.25	1.55	−1.48	−0.67	2.50	−1.75	−1.01	0.05	−0.25	1.10	−0.33
S_6_	3.15	−2.85	2.36	−0.02	−0.28	−1.79	0.03	2.05	1.88	0.15	−0.01	−2.05
S_7_	0.89	−0.04	1.25	−0.80	1.85	−1.33	−0.6	−0.01	−3.11	−0.08	−0.06	2.55
S_8_	0.22	0.89	0.04	0.32	2.35	−0.17	0.20	−1.18	−1.15	−0.25	−0.03	2.31
S_9_	−1.70	1.61	0.09	0.80	0.09	1.72	−1.03	−0.76	−1.88	−0.31	0.22	1.05
S_10_	−1.35	−0.16	−2.68	−0.03	−3.55	−0.06	−1.65	−0.14	−0.39	−0.07	−0.42	1.56
S_11_	2.92	−3.03	2.18	−0.25	−1.97	−0.15	−1.66	0.20	2.27	0.11	−0.06	−2.85
S_12_	3.11	−2.76	2.58	−0.19	−0.35	−1.58	1.27	0.01	1.95	0.09	−0.16	−2.45
S_13_	1.60	−0.12	−0.17	0.02	1.45	−1.77	−0.95	−0.90	−2.34	0.22	−0.15	2.90
S_14_	−0.95	2.15	0.62	1.58	−2.19	0.55	−1.05	−2.14	−0.66	−0.11	1.02	2.57
S_15_	−3.06	1.05	−2.28	1.95	−0.06	1.01	−1.44	2.13	2.85	−0.12	−1.15	−1.28
S_16_	−1.78	1.45	0.15	1.02	0.04	1.77	−1.12	−0.86	−1.89	−0.34	0.27	0.82
S_17_	0.25	0.91	0.01	1.44	2.55	−0.23	0.09	−1.26	−1.15	−0.29	−0.08	2.15
S_18_	1.66	−0.20	−0.11	0.08	1.25	−1.96	−0.95	−0.90	−2.12	0.40	−0.08	2.74

Indicator mean results are retained to two decimal places based on PAD scale benchmark values.

The actual measure of PAD for the target affective experience was calculated as shown in Equation 1 (32):


(1) $$\left\{ \begin{array}{c} P=\frac{C_{1}-C_{4}+C_{7}-C_{10}}{4}\\ \;A=\frac{-C_{2}+C_{5}-C_{8}+C_{11}}{4}\\ D=\frac{C_{3}-C_{6}+C_{9}-C_{12}}{4} \end{array}\;\right.$$

At the same time, some researchers (32, 37) have obtained the benchmark values of the 14 emotional characterizations of the PAD scale through comprehensive psychological experiments and computational analysis. Among them, the first 6 emotional characterization words belong to positive emotions, and the last 8 emotional characterization words belong to negative emotions, which can be calculated to determine the user’s affective response tendencies with their degree of inclination. This study determined the 8 emotion benchmark values applicable to the evaluation of the emotional experience of older adults, and the emotion characterization words of positive emotions were coded as 01–04, and the emotion characterization words of negative emotions were coded as 05–08, as shown in Table 4.

**Table 4:** PAD emotional characteristic benchmark value.

No.	Emotional characteristic	P_N_	A_N_	D_N_
1	Joy	2.77	1.21	1.42
2	Relaxation	2.19	−0.66	1.05
3	Surprise	1.72	1.71	0.22
4	Reliance	0.39	−0.81	−1.48
5	Boredom	−0.53	−1.25	−0.84
6	Fear	−0.93	1.30	−0.64
7	Anxiety	−0.95	0.32	−0.63
8	Anger	−1.98	1.10	0.60

P_N_, A_N_, and D_N_ are benchmark values for PAD emotional characteristics.

To further clarify the emotional experience tendency of the older adult users for each structure in the smart older care service system, the proximity between the actual measured values of the user’s emotional experience and the baseline value of the PAD was obtained through the Euclidean distance calculation (37). The smallest value of proximity for each of the eight emotional characteristics is the user’s experience tendency of the emotion of measurement index. The proximity is calculated by Equation 2.


(2) $$L=\sqrt{{\left(P-P_{N}\right)}^{2}+{\left(A-A_{N}\right)}^{2}+{\left(D-D_{N}\right)}^{2}}$$

Note: P_N_, A_N_, and D_N_ are all benchmark values for PAD emotional characteristics (37).

Substitute the PAD measurement values of each indicator in Table 3 into Equation 2, calculate the closeness between the PAD measurement values of each indicator and the reference values of 8 basic PAD emotional benchmarks, and obtain the multidimensional emotional experience tendencies of each indicator. The statistical results are shown in Table 5. The minimum value represents the corresponding sentiment of the indicator. From the statistical results, it can be seen that among the current 18 measurement indicators, 10 indicators are positive sentiments. The older users are relatively satisfied with the current smart care service system, but there are still some areas for improvement.

**Table 5:** PAD measurement results and emotional experience tendencies.

Code	PAD measurement value			PAD emotional experience tendencies							
				Positive emotion				Negative emotion			
	P	A	D	Joy	Relaxation	Surprise	Reliance	Boredom	Fear	Anxiety	Anger
S_1_	2.7050	1.6000	1.3600	0.3999*	2.3386	1.5106	4.3855	4.8402	4.1597	4.3540	4.7725
S_2_	1.9200	−0.6425	1.0000	2.0810	0.2751*	2.4865	2.9188	3.1236	3.8191	3.4381	4.2903
S_3_	−0.4500	0.4650	−0.5225	3.8336	3.2723	2.6096	1.8022	1.7460	1.6172	0.5316*	2.0010
S_4_	0.4300	−0.7525	−1.3350	4.1130	2.9655	3.1853	0.1610*	1.1892	2.5584	1.8846	3.6033
S_5_	1.7775	0.9775	0.4325	1.4192	1.7980	0.7649*	2.9628	3.4504	2.9300	3.0001	3.7632
S_6_	2.0950	0.1275	0.9425	1.3621	0.8005*	1.7796	3.1072	3.4591	3.6097	3.4325	4.2034
S_7_	0.7450	−0.6475	−1.4100	3.9446	2.8530	3.0274	0.3967*	1.5210	2.6817	2.1018	3.8104
S_8_	−0.2375	−0.9750	−0.8100	4.3350	3.0743	3.4788	0.9327	0.4026*	2.3841	1.4890	3.0545
S_9_	−1.0050	0.4750	−0.6000	4.3441	3.7708	3.1021	2.0908	1.8052	0.8294	0.1672*	1.6677
S_10_	−0.9600	1.2500	−0.5750	4.2302	4.0263	2.8330	2.6240	2.5505	0.0873*	0.9317	1.5632
S_11_	2.0950	0.9200	1.2375	0.7570*	1.8817	1.3417	3.6448	3.9894	3.5805	3.6221	4.1285
S_12_	2.1600	−0.6225	1.0375	1.9689	0.8189*	2.5105	3.0832	3.3399	4.0073	3.6525	4.5053
S_13_	0.3825	−0.7925	−1.4025	4.2044	3.0495	3.2686	0.0798*	1.1655	2.5851	1.9000	3.6295
S_14_	−1.015	0.4125	−0.5250	4.3296	3.7287	3.1175	2.0930	1.7602	0.8989	0.1543*	1.6339
S_15_	−2.0850	1.1600	0.7750	4.8979	4.6544	3.8844	3.8848	3.2916	1.8319	1.9919	0.2127*
S_16_	−1.0250	0.4975	−0.5250	4.3235	3.7625	3.0920	2.0656	1.8434	0.8162	0.2194*	1.5939
S_17_	−0.5225	−1.0325	−0.7725	4.5471	3.2890	3.6790	1.1759	0.2279*	2.3715	1.4256	2.9250
S_18_	0.4175	−0.7900	−1.3350	4.1381	2.9744	3.2194	0.1489*	1.1638	2.5820	1.8972	3.6145

Bolded data marked with * indicates the minimum value for this set of data, corresponding to the emotional characterization word in this column, indicating the user’s emotional experience tendency for the 18 indicators.

### 4.3 Emotional experience requirement assessment

After completing the determination of user emotional experience tendencies for each indicator, it is also necessary to analyze the level of user needs corresponding to each indicator. After secondary classification according to the Maslow’s hierarchy of needs theory, indicator task needs can be divided into positive demand, unactivated demand, and potential demand. The specific calculation method is as follows: Firstly, based on the initial questionnaire scoring results, calculate the average score of the sample sum for three parts: smart devices (indicators S_1_-S_5_), online software (indicators S_6_-S_11_), and offline services (indicators S_12_-S_18_), and use it as a benchmark. Secondly, use the standard deviation of each part as the criterion for judgment.

Finally, calculate the average score and standard deviation of each indicator. If the average score of the indicator is greater than the benchmark average score and the standard deviation is less than the benchmark standard deviation, it can be determined as a positive demand if the older respondents understand the content and express positive feedback clearly. The demand point where the average score of the indicator is less than the benchmark average score and the standard deviation is also less than the benchmark standard deviation is that the older respondents clearly use the content but do not necessarily express positive feedback, which can be judged as unactivated demand. The demand point where the average score of the indicator is greater than the benchmark average score and the standard deviation is greater than the benchmark standard deviation is that older users are unable to operate the indicator content and express negative feedback, which can be judged as potential demand.

The results of the demand determination are shown in Table 6.

**Table 6:** Result of emotional experience demand determination.

Code	Indicators	Average value	Standard deviation	Average > sum	Standard deviation < benchmark	Demand determination
	Smart devices	3.08	0.77	-	-	-
S_1_	Wearable health management devices	3.25	0.65	√	√	Positive demand
S_2_	Portable health monitoring devices	3.21	0.67	√	√	Positive demand
S_3_	Self-service medical check-up devices	2.97	0.72	×	√	Unactivated demand
S_4_	Home smart care monitoring devices	3.10	0.68	√	√	Positive demand
S_5_	Home service robots	2.88	0.75	×	√	Unactivated demand
	Online software	2.87	0.81	-	-	-
S_6_	Catering service apps	3.14	0.68	√	√	Positive demand
S_7_	Life service apps	2.98	0.75	√	√	Positive demand
S_8_	Shopping service apps	2.95	0.77	√	√	Positive demand
S_9_	Medical service apps	2.56	0.80	×	√	Unactivated demand
S_10_	Travel service apps	2.37	0.93	×	×	Potential demand
S_11_	Entertainment service apps	3.23	0.64	√	√	Positive demand
	Offline services	2.55	0.85	-	-	-
S_12_	Meal assistance services	2.83	0.65	√	√	Positive demand
S_13_	Life care services	2.76	0.67	√	√	Positive demand
S_14_	Mobility assistance services	2.31	1.02	×	×	Potential demand
S_15_	Home medical care services	2.44	0.74	×	√	Unactivated demand
S_16_	Offline medical care services	2.45	0.74	×	√	Unactivated demand
S_17_	Mental comfort services	2.39	0.93	×	×	Potential demand
S_18_	Emergency assistance services	2.68	0.67	√	√	Positive demand

According to the average results of the questionnaire indicators, the data is rounded to two decimal places.

## 5 Discussion

### 5.1 Visualization analysis of emotional experience evaluation results

**Figure 5:**
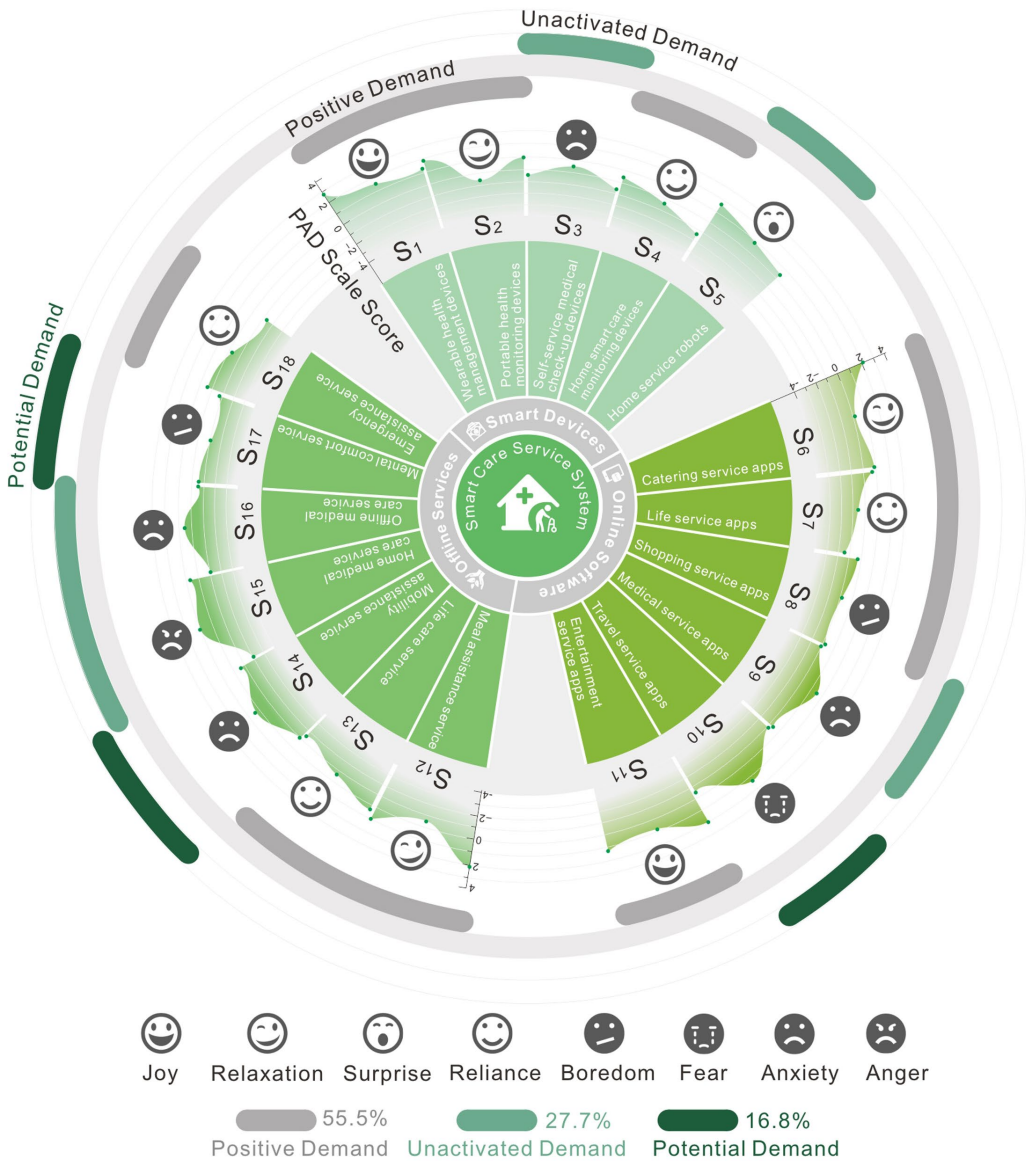
Visualization of analysis results.

As shown in Figure 5, the research team organized the results of the scale through visual analysis of graphics. We divided and summarized the touchpoints of smart devices, online software, and offline services into three parts. The PAD scale results were represented by a fan-shaped geometric structure, and the emotional experience tendency of each touchpoint was marked with emoticons. A bar structure was used to separate the results of the emotional demand judgment. It is easier for the visual graphics to determine which behavioral touchpoints should be experience-optimized.

According to the visualization results, a flat graph structure typically results in happy experiences, whereas the touchpoint behavior that creates negative experiences has a convex structure on the PAD quantification graph. A considerable percentage of negative feelings are experienced by older consumers when interacting with offline services and online applications. From the current emotional needs assessment results, positive demand accounts for the largest proportion, with 55.5%, unactivated demand accounts for 27.7%, and potential demand accounts for approximately 16.8%.

### 5.2 Emotional experience improvement strategy for smart care service system

The smart care service system consists of three main structures: smart devices, online software, and offline services. Based on the analysis of emotional experience data related to each part of the indicators, there are the following problems: from the analysis of older adults’ usage experience in smart devices, the percentage of pleasant emotions is significantly higher than that of negative emotions. The only occurrence of negative emotions in indicator S_3_ (self-service medical check-up devices), due to their going-out challenges, some older adults use self-service medical check-up devices seldom. Additionally, the equipment operation procedure lacks professional direction, which leads to ambiguous monitoring results and a lack of experience. Analyzing again from the aspect of emotional experience demand, indicators S_3_ and S_5_ (home service robots) are all in the category of unactivated demand, indicating that self-service medical check-up devices and home service robots are less used in the process of aging, but older adults are still interested in the use of this type of equipment, only that under the influence of objective factors, this type of equipment has not become a mainstream support product for aging services.

From the analysis of the usage of online software by the older users, the proportion of positive emotions is the same as that of negative emotions. The generation of negative emotions is indicated by indicators S_8_ (shopping service apps), S_9_ (medical service apps), S_10_ (travel service apps). Older adult consumers’ buying habits are more reliant on in-person purchases, and they have historically used internet shopping software at a low rate. Furthermore, older adults face additional difficulties and pitfalls due to the sophisticated and varied internet purchasing software. Older adults are still relatively accustomed to using online medical software, but the process of obtaining medical attention stresses them out and makes them nervous. However, the use of online medical software can provide medical services with a certain level of convenience that corresponds to their passive emotional demands. Older adults use travel service software at a very low rate, and the majority of them are afraid because they find it impossible to go on their own. Nonetheless, there is a certain amount of travel necessity, which could lead to a desire for this kind of software from older adults who have not used it yet but would like to.

Based on the experience evaluation of the older users toward offline services, the proportion of negative emotions is higher than that of positive emotions. The indicators for experiencing negative emotions are S_14_ (mobility assistance services), S_15_ (home medical care services), S_16_ (offline medical care services), S_17_ (mental comfort services). In terms of the service experience of helping older adults travel, there are currently few professional service personnel in this area, which has failed to effectively meet the travel needs of older adults, resulting in a certain degree of anxiety, travel needs are also potential needs for them. The main issue with home medical care services is that there are not enough expert staff available to offer medical and health care on-site. Older adults in emergencies frequently become resentful because home-based services for early treatment have not yet been developed to their full potential. Additionally, there is a growing need for this service. For the experience of offline medical care services, older adults participate in health consultations and knowledge lectures. Rehabilitation training equipment is inferior to that of professional medical facilities, and senior citizens’ mental health is another important issue that warrants consideration. Therefore, there are unactivated demand points for offline medical care services. Regarding spiritual comfort services for older adults, it is equally important. Research shows that older adults who can participate in community activities, academy for older adults, and other entertainment projects are psychologically healthy, while those who lack recreational activities exhibit negative emotions.

Synthesizing the above discussion, smart devices are currently the fastest-growing category of smart older adult care products. Compared to virtual electronic services, smart devices have a much higher overall application rate. However, due to the complexity and diversity of these products, there are discrepancies in emotional responses during the user experience of older users. Our research has made efforts to standardize classifications as much as possible, but there are still some differences in product experiences. This provides areas for improvement in our future research on refining evaluation criteria. Online software also exhibits complex and diverse characteristics, and its update and replacement cycle is faster compared to smart devices. Currently, older users’ demand for offline services remains relatively stable. Guided by these insights, we propose the following improvement strategies for the smart care service system.

#### 5.2.1 Emotional experience improvement strategies for smart devices

##### 5.2.1.1 Promote the operation of smart devices

According to the emotional experience evaluation results of older users toward smart devices, the overall satisfaction can meet the expectations of older users. Strong community and service staff collaboration is required to sustain older users’ excitement for using various health management devices. To prevent older adults from resisting, disliking, or being concerned about smart devices, more people should be made aware of how the devices work. To create a digital health service system for older adults and improve service direction, smart devices can gather health data from older adults within a suitable range and without infringing on personal privacy. The promotion breadth and aging compatibility of products that may be in demand from older adults, including indicators S_3_ (self-service medical check-up devices) and S_5_ (home service robots), should be increased. Additionally, expert operators should be introduced to offer prompt instruction to older consumers in everyday services. Health lectures and on-site testing can be used to expand the scope of promotion.

#### 5.2.2 Emotional experience improvement strategies for online software

##### 5.2.2.1 Establish an older-adult-friendly cloud service platform

According to the usage of online software by older adults, they are currently able to apply basic life security software types well. However, using functional software can lead to negative emotions. The main cause is that functional software is hard to use for older adults. The channels available to the older adults for obtaining different service information are limited and lagging, and there are numerous software types as well. To ensure that older adults maintain the operational enthusiasm of the touchpoint indicators S_6_ (catering service apps), S_7_ (life service apps), and S_11_ (entertainment service apps) while improving negative emotions in other indicators, it is proposed to establish an older-adult-friendly cloud service platform. Different demand service function modules are separated based on the examination of the older adults’ behavior touchpoints in emotional evaluation measurement. Big data is used to build an application platform with easy-to-use information and convenient services for the older population, as well as to construct an older-adult-friendly medical and older adults’ care cloud service system. Integrating current software for older adults’ care services, progressively integrating different features, and making it more appropriate for older adults operations are the goals.

#### 5.2.3 Emotional experience improvement strategies for offline services

##### 5.2.3.1 Integrate relevant stakeholder resources

In the present smart care service system, older adults are generally not particularly satisfied with offline services. There are numerous comprehensive causes, including dispersed community resources, a lack of links among pertinent stakeholders, and social resources that are on the periphery of services and only infrequently engage with certain older populations. Older adults are more reliant on their children’s care, have a lower percentage of mutual assistance in the system, and feel more anxious when participating in activities that take place outside of their homes. To better promote the implementation of the older-adult-friendly cloud service platform, more social forces, including stakeholders related to older adults’ care services, need to participate. In terms of indicators S_13_ (life care services) and S_16_ (offline medical care services), in recent years, emerging professions such as bathing assistants and accompanying therapists have gradually emerged in the older adults’ care industry, flexibility and diversity of services are constantly being stimulated. Service design aims to actively promote the growth of the older adults’ care industry, plan service resources at all levels, integrate relevant stakeholders systematically, provide participation paths for various resources to enter the smart care service system, and supply multiple service models for those rich older care needs. For indicator S_17_ (mental comfort services), it is necessary to attract more service professionals to enter the smart older adult care service system to increase the possibility of public participation in the implementation of older adults’ care service paths and further promote the construction of multi-resource collaborative digitization.

## 6 Conclusion

Based on the PAD emotion theory and guided by service design research methods, our study applied service scenario interaction touchpoint maps to expand evaluation dimensions and establish an emotional experience evaluation index system for the smart care service system. Through experimental process of emotional experience evaluation and measurement, on the one hand, we obtained the value of the pleasure-arousal-dominance emotions of the older users for the measurement indexes, and derived the tendency of emotional experience through calculation. On the other hand, according to Maslow’s hierarchy of needs theory for secondary classification, emotional experience needs were determined for each indicator after calculation, including positive demand, unactivated demand, and potential demand. The results for the evaluation of emotional experience were derived from the measuring experiments. The assessment findings of the smart devices, online software, and offline services of the smart care service system were examined using visual classification analysis techniques, and pertinent ideas for enhancing the emotional experience of each component were suggested. This work extended the multidimensional evaluation experimental approach for evaluating emotional experiences. By quantifying data, it can better determine the shortcomings of the existing smart care system and propose strategies for improvement. Our research team will continue to refine techniques for assessing the emotional experiences of older adults while investigating new service models offered by the older adults’ care industry. Our work aims to provide this demographic with more diverse and practical options, ease the social pressures associated with their care, and encourage the robust growth of this sector.

## Data Availability

The original contributions presented in the study are included in the article/supplementary material, further inquiries can be directed to the corresponding author.
